# Assessing the Mechanical Weakness of Vertebrae Affected by Primary Tumors: A Feasibility Study

**DOI:** 10.3390/ma13153256

**Published:** 2020-07-22

**Authors:** Marco Palanca, Luca Cristofolini, Alessandro Gasbarrini, Giuseppe Tedesco, Giovanni Barbanti-Bròdano

**Affiliations:** 1 Department of Oncology and Metabolism and INSIGNEO Institute for in Silico Medicine, The University of Sheffield, Pam Liversidge Building, Sheffield S1 3JD, UK; 2 Department of Industrial Engineering, School of Engineering and Architecture, Alma Mater Studiorum—Università di Bologna, Via Terracini 24–28, 40131 Bologna, Italy; luca.cristofolini@unibo.it; 3 Department of Oncological and Degenerative Spine Surgery, IRCCS Istituto Ortopedico Rizzoli, Via G.C. Pupilli, 1-40136 Bologna, Italy; alessandro.gasbarrini@ior.it (A.G.); giuseppe.tedesco@ior.it (G.T.); giovanni.barbantibrodano@ior.it (G.B.-B.)

**Keywords:** vertebra, spine disease, primary tumor, in vitro tests, strain analysis, mechanical stability, digital image correlation

## Abstract

Patients spend months between the primary spinal tumor diagnosis and the surgical treatment, due to the need for performing chemotherapy and/or radiotherapy. During this period, they are exposed to an unknown risk of fracture. The aim of this study was to assess if it is possible to measure the mechanical strain in vertebrae affected by primary tumors, so as to open the way to an evidence-based scoring or prediction tool. We performed biomechanical tests on three vertebrae with bone tumor removed from patients. The tests were designed so as not to compromise the standard surgical and diagnostic procedures. Non-destructive mechanical tests in combination with state-of-the-art digital image correlation allowed to measure the distribution of strain on the surface of the vertebra. Our study has shown that the strains in the tumor region is circa 3 times higher than in the healthy bones, with principal strain peaks of 40,000/−20,000 microstrain, indicating a stress concentration potentially triggering vertebral fracture. This study has proven it is possible to analyze the mechanical behavior of primary tumor vertebrae as part of the clinical treatment protocol. This will allow building a tool for quantifying the risk of fracture and improving decision making in spine tumors.

## 1. Introduction

Primary malignant spinal tumors consist of a large spectrum of various histologic entities. Osteosarcoma is the most frequently diagnosed (35.1%), followed by chondrosarcoma (25.8%) and Ewing’s sarcoma (16.0%) [1], and compressively they represent the 0.2% of all malignant tumors. The overall 5 years survival rate is around 68%, when proper and effective treatments are identified and applied [2]. Conversely, severe complications could occur and, in addition, the medical case can become more difficult.

Indeed, a multidisciplinary approach between medical oncologists, radiation oncologists and spine surgeons is mandatory for choosing the appropriate treatment and timing of treatment. Usually, patients with a diagnosis of a primary spine tumor undergo chemotherapy and/or radiotherapy to diminish osteoclasts activations and kill the tumor cell, shrinking the tumor volume and producing ossification [3]. During the weeks/months of therapy, before surgical intervention, the patient is exposed to the risks of fracture. From an orthopaedic point of view, a careful evaluation of spinal stability is crucial in the decision-making process: in some cases, over-protection may result in unnecessary constraint and reduction of quality of life, while insufficient treatment may lead to fractures and deformities of the spine with possible neurological sequelae. Moreover, the timing of the planned surgery, connected with the progression of the pathology, can play a fundamental role in the reduction of the risk of fracture.

Similar problems are experienced by patients with metastatic spine diseases, where surgical treatment decisions are broadly based on spinal stability and patient-specific factors that include patient health, prognosis, and tumor histology [4,5]. In 2010, the Spine Oncology Study Group (SOSG) introduced a classification system for spinal instability in neoplastic disease (SINS) [4], which takes into account six different factors: location of the tumor within the spine, mechanical pain, bone lesion quality, spinal alignment, vertebral body collapse and posterolateral involvement of spinal elements. The SINS is generated by tallying each score from the six individual components. The minimum score is 0, and the maximum is 18. Scores up to 6 denote a “stable” spine, scores between 7 and 12 denote “indeterminate (possibly impending) instability”, and scores greater than 13 denote “instability”. According to the SOGS study, SINS scores between 7 and 18 warrant surgical consultation. The SINS score has proved to be an excellent tool for non-surgeons to identify the patients that should be referred to a spine surgeon, with an excellent inter-observer and intra-observer agreement [6,7]. However the SINS score is only partially helpful for surgeon as a treatment guide [8]: there is a large grey zone of “potentially unstable” (SINS 7 to 12), were spine surgeons currently must rely on their own experience to determine whether instability is present in the setting of spinal neoplasia and surgery is urgently indicated. A recent review [9] confirmed that the recommendations for treating the primary bone tumors are based on moderate quality evidence and expert opinions.

To assist surgeons in the decision-making process, several biomechanical studies have shown that the modifications associated with simulated metastatic lesions (and the consequent risk of instability) are to a large extent related to biomechanical factors [10,11,12]. In fact, vertebral fracture in case of tumors is possibly triggered by the limited mechanical strength of neoplastic tissue, and by the stress concentrations occurring around tumor lesions. Therefore, the paradigm for investigating spinal instability as the result of a neoplastic process differs significantly from traumatic injuries under different points of view: the pattern of bony and ligamentous involvement, the prognosis, neurologic manifestations and bone quality. Specific and different sets of criteria for assessing spinal stability are required. In particular, a biomechanical path for proving the evidence of the effects of primary malignant tumors on the spine stability has not yet been defined.

The hypothesis of this study was that the primary tumors cause significant mechanical alterations, in terms of bone strain distribution and absolute value in the spine. The aim was to verify if such alterations can be measured in vitro in order to define a reliable way to characterize the effect of the specific tumors. In prospective, this approach can be used to collect a database from a range of tumors, and create an evidence-based score to assess the stability of the spine.

## 2. Materials and Methods

### 2.1. Protocol

**Figure 1:**
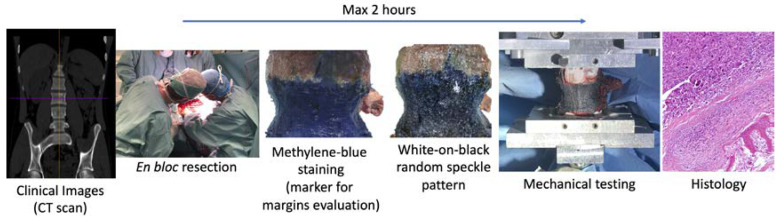
The workflow consisted of the CT-scan of the patient, en bloc resection of the vertebra with primary tumor, staining of the vertebra with methylene blue for the evaluation of the tumor margins, preparation of the white-on-black random speckle pattern for the DIC measurements, mechanical tests and histology. The time available between the resection of the vertebra and the end on the mechanical test was two hours for not jeopardizing the patient’s diagnosis.

The workflow (Figure 1) was defined in compliance with the rules of the Declaration of Helsinki and the protocol was approved by the Ethical Committee of the Rizzoli Orthopaedic Institute (Protocol 441, 15/01/2018, Bologna, Italy); written informed consent was provided by all the patients involved.

After the en bloc resection of the primary tumor, the retrieved spine specimen can be used for biomechanical tests, with two strong restrictions:

- The specimen can stay in air only two hours to not modify the biological characteristics, after that the specimen must be fixed in formalin solution (also if the tests are not completed);
- The specimen must not be fractured or damaged to not compromise the evaluation of the margins of the tumor and the histological analysis.

The design of the biomechanical test was constrained by the need for not changing the clinical pathway, and not jeopardizing the diagnosis based on the histology of the removed tissue.

### 2.2. Cases

Three cases were collected for this study at the Rizzoli Orthopaedic Institute in Bologna, Italy. The selection criteria were chosen to widen the range of applicability to different clinical scenarios and, at the same time, guarantee the optimal metrological conditions. For these reasons, only vertebrae with a large vertebral body (generally from T10 to L5), without any fracture signs, without any history of spinal surgery and spinal fixation and from non-osteoporotic donors were accepted. The batch had to include vertebrae treated with, and one without, radiotherapy. The patient’s details are reported in Table 1.

**Figure 2:**
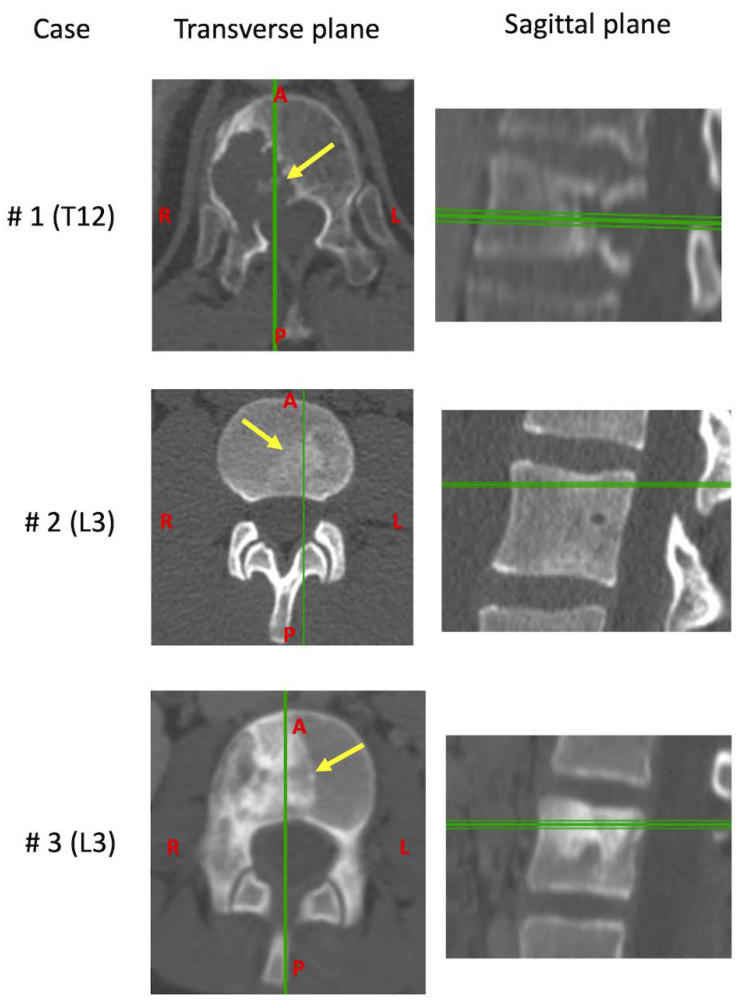
Transverse and sagittal planes of the CT scans for each vertebra before the resection. The arrows indicate the position of the tumor, the vertical and horizontal lines indicate the relative position of the plans.

Surgeries were planned and performed according to the location of the lesion in order to minimize the risk of both neurological lesions and marginal contamination. Clinical CT scans were performed the day before the surgery (Figure 2).

In the first case (#1), the vertebrectomy was performed through a posterior approach and the resection was achieved through the disc above and below. In the second case (#2), a double approach (initially posterior and then combined posterior and anterior extra-pleural retro peritoneal approach) was performed and the resection was achieved through the cranial and caudal adjacent vertebral bodies. In the third case (#3) a double approach similar to specimen #2 was performed and the resection was achieved through the cranial and caudal adjacent discs.

In all cases, the posterior arc was removed and the dural sac was protected with the Chiripa technique [17]. In cases #1 and #2, the osteotomies were performed with the Gigli saw and the Resegone retractor (K2M, Leesburg, VA, USA); in case #3 the discectomy was performed with forceps.

At the end of the surgery, x-ray images of the resected vertebral body were acquired and the bone integrity was checked. Thus, the vertebral bodies were sent to the department of pathological anatomy for the margin evaluation, then to the biomechanics lab for the mechanical tests. Finally, the specimens were sent back to the hospital for the histological analysis.

### 2.3. Mechanical Tests

The specimens were aligned with a well-established anatomical reference frame to guarantee the repeatability of the measurements [18] and the extremities were potted in bone cement.

**Figure 3:**
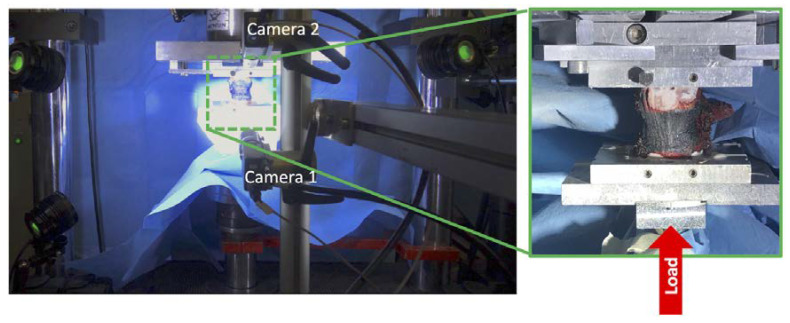
Left: loading setup with 3D-DIC cameras and light system. The low friction linear bearings and the ball joint are covered by the blue towels. On the right, a detail of the specimen with the speckle pattern (anterior view).

The tests were performed using a uniaxial testing machine (Instron 8032 with the Instron 8800 controller, Instron, Cambridge, UK) and a 10 kN load cell (Instron, Cambridge, UK). Each specimen was fixed on the caudal side and compressed from the cranial side. To generate presso-flexion [11,19], the compression force was applied with an anterior offset of the 10% of the antero-posterior dimension of the vertebral body with respect to the center of the vertebral body. To avoid transmission of any undesired component of load (simulate the role of the intervertebral discs), free rotation of the cranial pot was allowed by means of a ball joint, while free horizontal translations were granted by means of two low-friction orthogonal linear bearings (Figure 3). The specimen was loaded initially with a very small load of 50 N so as to preliminarily check the strain distribution was performed, and the average strains were computed. Based on this pre-tuning of the load, the actual load was computed so as to reach an expected average compressive strain of 1500 microstrain (which is the strain magnitude associated with physiological loading conditions) on the anterior surface of the vertebral body [20,21,22]. The tests were performed with extreme caution to ensure that no damage was induced in the specimen that would compromise the diagnosis for the patients. In fact, in case of load drop, indicating a fracture, the entire test would have been immediately stopped (this event did not occur in any specimen).

### 2.4. Strain Measurements

Before the test, a white-on-black speckle pattern [23,24,25] was prepared on the anterior surface of the vertebral body to measure the strain on the anterior cortical surface with a validated three-dimensional Digital Image Correlation [11,25]. Briefly, the dark background was prepared by staining the anterior surface of the vertebral body with a saturated solution of methylene blue; this solution does not change the mechanical properties of biological specimens [23,25]. The white speckle dots were obtained spraying a water-based white paint (Q250201 Bianco Opaco, Chrèon, Como, Italy) with an airbrush airgun (AZ3 HTE 2, nozzle 1.8 mm, Antes Iwata, Italy).

The 3D-DIC system (Q400, Dantec Dynamics, Skovlunde, Denmark) used was equipped with two cameras (5MPixels, 2440 × 2050, 8-bit, Stingray F-504b, Allied Vision, Stadtroda, Germany) with metrology-standard 35 mm lenses (Apo-Xenoplan 1.8/35, Schneider-Kreuznach, Bad Kreuznach Germany; 135 mm equivalent), and a custom set of LEDs, with a luminous flux of 10,000 lumen. To optimize the field of view and the resolution, the cameras were vertically positioned in front of the specimen (Figure 3). The distance between the cameras and the specimen was adjusted for each vertebra to optimize the framing, obtaining an averaged pixel size of 25 µm. Images were acquired at 10 frames per second during the load application, with the lens aperture equal to f/16 and a shutter time of 1/50 s.

The tensile and compressive strains on the anterior surface of the vertebral body were computed with Istra 4D (v.4.3.1 Dantec Dynamics) using a facet size in the range 45–49 pixels, a grid spacing between 17 and 30 pixels, and a filter kernel size with 11 × 11 measurement points. This combination of optimized parameters [26] allowed a measurement spatial resolution below 3 mm and the minimization of the noise on the measurements.

Finally, in order to provide a quantitative estimate of the damage extent caused by the tumor, the ratio between the average principal strains on the surface in front of the tumor and the average principal strain on the vertebral body were computed. In fact, in a healthy vertebra, a uniform strain distribution is expected [21], while in case of tumor lesions strain concentrations could occur [11].

## 3. Results

To better understand the detailed strain analysis below, the reader should bear in mind two points: (1) the geometry and material properties of healthy vertebrae are optimized for their daily loads so as to provide a uniform distribution of strain. In fact, differences of less than 5% were measured in vitro around the vertebral bodies of healthy vertebrae in axial compression [21]. While a uniform strain distribution corresponds to and optimized structure and a reduced risk of fracture, the opposite happens when high strain peaks occur for some reason (e.g., tumor lesions). (2) Under physiological loading, the strain experienced by bone tissue is the range 2000/−2000 microstrain [27]. When strains exceed 7000/−10,000 microstrain, damage occurs in healthy cortical bone [28].

The maximum load was tuned for each specimen so as to reach an average compressive strain of 1500 microstrain (which is the strain magnitude associated with physiological loading conditions). To achieve such strain magnitudes, the loads (force applied with a 10% anterior offset) for the three specimens were, respectively, 214, 397 and 191 N.

**Figure 4:**
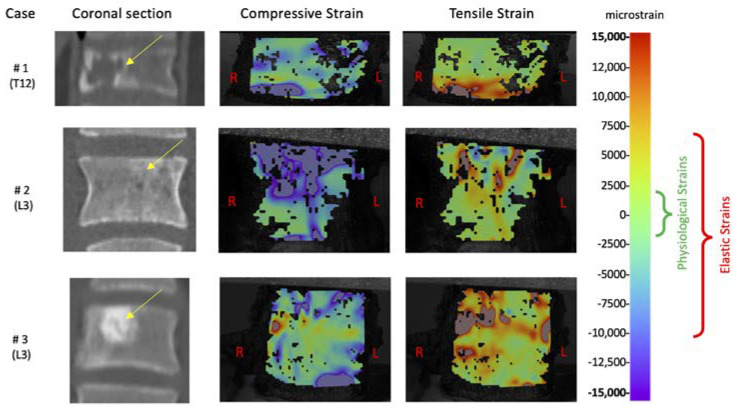
Compressive and tensile strains experimentally measured on the vertebral anterior surface. The coronal sections show the position of the tumor (arrows). The false color map represents in reddish color the tensile strain, and in bluish color the compressive strain. The green bracket indicates the physiological strain range, the red bracket indicates the elastic strain range for healthy cortical bone [20,28].

The tensile and compressive strains were analyzed in detail under such maximum load. All specimens showed a highly inhomogeneous strain distribution on the anterior surface of the vertebral body (Figure 4). The average compressive strains were around 1500 microstrain, as described above, but the tensile peak strains reached 40,000 microstrain while the compressive peak strain were −20,000 microstrain. In all three specimens, the strain peaks on the anterior surface of the vertebral body were in correspondence of the tumor. The ratios of the principal strains between the region in front of the tumor and the vertebral body were, for the tensile strain: 3.2 (#1), 1 (#2) and 2.7 (#3), and for the compressive strain: 1.8 (#1), 1.7 (#2) and 3 (#3).

## 4. Discussion

This work aimed to verify the feasibility of investigating by means of a biomechanical analysis the reduction of spinal stability in case of primary tumor, in order to create a reliable tool applicable to obtain evidence. En bloc resected vertebrae were subjected to mechanical testing and the altered strain distribution due to the tumors was measured. The results showed that for the tested specimens with primary tumor, the spine was largely unstable. Indeed, the specimens were tested with loads lower than the ones associated with typical physiological activities but an alarming strain distribution with strain peaks over the typical failure limit (circa 6-fold the typical tensile fracture strain and twice the typical compressive fracture strain, which are 7000 microstrain in tension and −10,000 microstrain in compression [20,28]) were measured.

The comparison with the strain distribution in healthy vertebrae [11,21] revealed that stability was influenced by the presence of the tumor both in terms of absolute strain and in terms of strain distribution. In particular, the qualitative strain analysis highlighted the vertebral weakness in correspondence of the tumor mass. This suggested that a slight misalignment of the load, during daily activities, could result in a vertebral fracture, worsening the medical case. At the same time, despite the strain values were over the typical bone fracture threshold, the vertebrae did not show any sign of fracture. This behavior indicated the regions in correspondence of the tumor had also different mechanical properties that micro-/nano-indentation tests can confirm [29,30].

In the literature, some works evaluated the strain on vertebrae affected by metastasis but, to the best of the authors’ knowledge, this is the first case in which the strain distribution was measured in vitro on en bloc resected vertebrae, and generally other bones, with primary tumor. From a biomechanical point of view, several aspects are similar in the case of primary and secondary tumors (metastasis). The presence of a lytic mass inside the vertebra causes a reduction of the vertebra strength and, indirectly, increases the risk of clinically observed fractures [31,32,33]. This increased risk of facture is due to a dramatic alteration of the strain pattern with respect to the naturally optimized structure of the healthy vertebra [11,34,35], with strain peaks above the typical yield strain of healthy bone.

The lack of evidence in the biomechanical field of the effects of the primary tumors on the spine directly impinges on the clinical practice: clear guidelines are currently missing to face the dilemma between therapy and surgery in case of primary tumor, both benign and malign such as Ewing’s sarcoma, chondrosarcoma, giant cell tumor, hemangioma, osteoblastoma, etc. Indeed, treatment decision is still based predominantly on retrospective case series and institutional expertise [36]. A better scenario, instead, is available for the treatment of spinal metastasis, where the SINS [4], the Tomita [37] and the Tokuhashi classifications [5] provide basic guidelines for the clinicians. However, the indications from these classifications are conceived for secondary tumors and cannot be directly extended to primary ones. Only the SINS can provide this sort of information in the case of primary tumors. However, in two cases of this study (#2 and #3, with SINS scores = 4 and 6, respectively) the SINS would have suggested no spinal stabilization, and only in one case (#1, SINS score = 11) consultation with a spine surgeon would have been recommended, while our biomechanical evidence highlighted that all three cases were highly at risk of fracture.

The main limitations of this study were due mainly to the rarity of the pathologies (0.2% of all malignant tumors [2]) and the need not to jeopardize the diagnosis for the patients, as imposed by the ethical committee for this pilot study. First of all, the number of specimens, the kind of tumors and the intrinsic biological variability did not allow to generalize the clinical outcomes of the study. Despite the results being consistent, other tests must be performed to build a statistically robust body of information. The study was performed to define a reliable path applied to vertebrae with different conditions, including different types of tumor, but also subjected or not subjected to radiotherapy (which is known to affect the mechanical behavior of the bone [38]), to verify the ability of capturing the effect of the tumor. A description of the vertebra behavior up to failure and of the strain inside the vertebral body were not allowed, but they would help to understand which part of the vertebra is largely stressed. Moreover, the results invite to perform a micro-/nano-indentation analysis for the different kind of tumors to characterize the tumor tissue and understanding the role played in weakening the vertebra.

In this paper, we presented how the proposed biomechanical test can be used to investigate the alterations associated with bone tumors. This approach can be extended and applied in different contexts. For instance, breast tumor is known to affect the stiffness of the extracellular matrix which, in turns, modulates the response of tumor cells [39,40]. Therefore, also in this area, investigating the alterations of the strain distribution could provide useful information for the diagnosis and/or treatment [41].

In conclusion, this work showed the feasibility of assessing the biomechanical effect of vertebral primary bone tumors by measuring the strain distribution on the anterior surface of en bloc resected specimens. The strain pattern showed the specimens were highly unstable due to the presence of the tumor, and close to failure even with loads lower than the physiological ones. This study could raise awareness among the surgeons in taking care of the balance between a proper drug treatment for the tumor and the urgency of a spine stabilization to reduce the risk of fracture. This work could pave the way to the idea of identifying an assessment, based on evidence, that takes into account both the clinical condition of the patient and the biomechanical condition of the spine.

## Figures and Tables

**Table 1:** Patients’ and tumors’ details. In particular, for each case, the following details were evaluated: surgical stage (Enneking Classification) [13], tumor extension (WBB Classification) [14], epidural spinal cord compression (Bilsky Classification) [15], spine instability neoplastic score [4], and margin classification (and for the Ewing’s sarcoma also the prognostic indicator [16]).

Case	Age	Sex	Tumors	Vertebra	Grade	Local Extensions	6-Point ESCC	SINS	Margins Classification	Presence of Metastasis	Months between Diagnosis and Surgery	Previous Therapy	Current Therapy
#1	62	M	Chordoma	T12	IB	7–10	1B	11	Focal margin, in the posterior part of the specimen. Wide, the other margins	NO	2	None	None
#2	22	M	Ewing’s sarcoma	L3	IIB	2–6	0	4	Wide (Necrosis 40%, Bologna system 1)	NO	4	Chemotherapy	Chemotherapy and Radiotherapy
#3	11	F	Ewing’s sarcoma	L3	IIB	7–11	1A	6	Wide (Necrosis 80%, Bologna system 1)	NO	6	Chemotherapy	Chemotherapy and Radiotherapy
